# Genome sequence and plasmid transformation of the model high-yield bacterial cellulose producer *Gluconacetobacter hansenii* ATCC 53582

**DOI:** 10.1038/srep23635

**Published:** 2016-03-24

**Authors:** Michael Florea, Benjamin Reeve, James Abbott, Paul S. Freemont, Tom Ellis

**Affiliations:** 1 Department of Life Sciences, Imperial College London, London, UK; 2 Centre for Synthetic Biology and Innovation, Imperial College London, London, UK; 3 Bioinformatics Support Service, Department of Surgery and Cancer, Imperial College London, London, UK; 4 Centre for Integrative Systems Biology and Bioinformatics, Imperial College London, London, UK; 5 Department of Bioengineering, Imperial College London, London, UK; 6 Department of Medicine, Imperial College London, London, UK

## Abstract

Bacterial cellulose is a strong, highly pure form of cellulose that is used in a range of applications in industry, consumer goods and medicine. *Gluconacetobacter hansenii* ATCC 53582 is one of the highest reported bacterial cellulose producing strains and has been used as a model organism in numerous studies of bacterial cellulose production and studies aiming to increased cellulose productivity. Here we present a high-quality draft genome sequence for *G. hansenii* ATCC 53582 and find that in addition to the previously described cellulose synthase operon, ATCC 53582 contains two additional cellulose synthase operons and several previously undescribed genes associated with cellulose production. In parallel, we also develop optimized protocols and identify plasmid backbones suitable for transformation of ATCC 53582, albeit with low efficiencies. Together, these results provide important information for further studies into cellulose synthesis and for future studies aiming to genetically engineer *G. hansenii* ATCC 53582 for increased cellulose productivity.

Bacterial cellulose is a strong and pure form of cellulose that is produced in large quantities by species of *Acetobacteraceae*, possibly due to the benefits of cellulose in food colonization and protection against environmental hazards^1^,^2^. Cellulose production is catalysed by AcsAB from the *acs* (*Acetobacter* cellulose synthase) operon, which incorporates glucose monomers into growing cellulose fibrils using UDP-glucose as a substrate^3^. Cellulose fibrils are then secreted through an outer membrane pore formed of AcsC^2^. *Acs* operon also contains *acsD*, which localizes into the periplasm and has been implicated to be important in crystallization of glucan chains^2^. Cellulose synthesis seems to be regulated based on environmental signals, as the activity of AcsAB is controlled by the second messenger cyclic dimeric guanosine monophosphate (c-di-GMP), which activates AcsAB and is required for cellulose synthesis^4^. In materials science, bacterial cellulose has been a focus of research due to its several unique properties: unlike plant-based cellulose, it is free of contaminating chemical species such as lignin and pectin, and is synthesized as a continuous interconnected lattice^2^. It is over 15 times stronger than plant-based cellulose (bacterial cellulose has a tensile strength over 1500 MPa compared to 100 MPa of plant cellulose)^5^, and is biocompatible, flexible and capable of storing water more than 10 times its own weight^6^,^7^. Due to these qualities, it is used commercially in high-quality acoustic speakers, medical wound-dressings, health foods, and other products^2^. However, potential applications of bacterial cellulose are much more wide-ranging than its current industrial uses, as it can be used to create biocompatible, artificial tissue scaffolds and blood vessels^7^,^8^, flexible electrodes and OLED displays^9^, sensors^10^ as well as other materials^11^.

From the different *Acetobacteraceae* species and strains, *G. hansenii* ATCC 53582 has been reported as a high-yield cellulose producing strain^12^,^13^, has been used in numerous studies into the genetics, biochemistry, mechanical properties and increased production yields of bacterial cellulose^13^,^14^,^15^,^16^,^17^,^18^,^19^, and has thus become an important model organism for studies of bacterial cellulose production over the past three decades. So far, studies aiming to increase bacterial cellulose production have mainly focussed on optimizing culturing conditions^2^,^11^, however these have or will necessarily reach limits determined by the genetics of the producing bacterium. A few attempts at genetic engineering has been made using lower yield cellulose producing *Gluconacetobacter* strains^20^,^21^, however to achieve increased cellulose production it is desirable to use ATCC 53582 or another already high cellulose producing strain as the platform. We have sequenced the genome and developed protocols for transformation of *G. hansenii* ATCC 53582. From the genome sequence, we identify several new genes associated with cellulose synthesis. We also identify several plasmid backbones capable of replication in ATCC 53582, offering a starting point towards future genetic engineering of this strain.

## Results

### Genome sequencing

**Figure 1:**
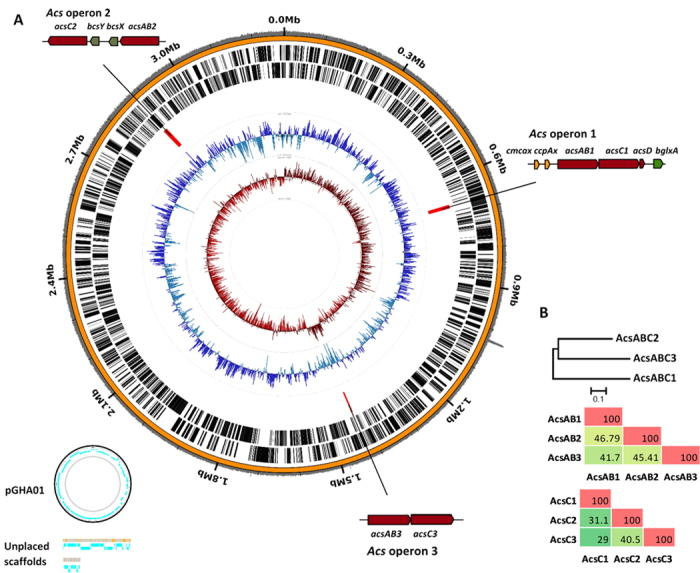
Overview of *G. hansenii* ATCC 53582 genome. **(A)** *G. hansenii* ATCC 53582 genome is 3.27 Mbp in size, with a GC% of 59.48, and contains a predicted 2988 protein coding genes, 49 tRNAs, 1 tmRNA, and 7 rRNA genes. The genome contains a chromosome of approximately 3.27 Mbp and at least one 51 kb plasmid pGHA01. Additionally, it contains 2 scaffolds (40 kbp, 10 kbp), which could not be confidently placed. The chromosome contains the previously described *acs1* operon, and two additional, undescribed *acs* operons (*acs2*, *acs3*), which differ from each other in gene content. For the chromosome, rings show from outside in: (1) read coverage, (2) coding sequences on forward and (3) reverse strands, (4) *acs1*, *acs2* and *acs3* operons, with their gene contents magnified, (5) GC percentage and (6) GC skew. Read coverage was similar across the genome (see outer ring), with an average RPKM value of 477. See Materials and Methods for details on sequencing and analysis. **(B)** Relatedness and amino acid percentage identity of AcsAB and AcsC proteins in the three *acs* operons. A phylogeny of AcsABC proteins suggests *acs2* and *acs3* to be more closely related to each other than the full *acs1* operon. Furthermore, as *acs2* and *acs3* do not contain *acsD*, *cmcax, ccpax* nor *bglxA,* this suggests that they may have arisen in evolution as duplications of *acs1*, followed by gene loss. AcsAB proteins seem to be more conserved than AcsC, and share approximately 41–46% sequence identity, compared to 29–40% identity of AcsC. Amino acid sequences were aligned and percent identity calculated using MUSCLE^54^ and the tree built using the Neighbour-Joining method. All positions containing gaps were removed from analysis. Analyses were conducted using MEGA6^55^.

We sequenced *G. hansenii* ATCC 53582 to approximately 800x coverage using Illumina MiSeq 250 bp paired reads and assembled the reads using the BugBuilder pipeline^22^ (ENA submission ID PRJEB10804). We tested scaffolding using *G. xylinus* E25^23^, *G. hansenii* ATCC 23769^24^, and *G. xylinus* NBRC 3288^25^ as reference genomes, and chose the NBRC 3288, as this resulted in the best overall scaffold with fewest unplaced contigs and incorrect rearrangements. The resulting scaffolds were then manually checked and edited for quality control. Sequencing revealed that the genome of ATCC 53582 is 3.37 Mbp in size with a GC% of 59.48, and contains 2988 protein coding genes, 49 tRNAs, 1 tmRNA, and 7 rRNA genes (of which 4 are partial 5S rRNAs) (Fig. 1). The genome could be assembled into a chromosome of 3.27 Mbp, a large plasmid of 51.3 kbp (pGHA01), and two unplaced scaffolds (40.1 kbp, 10.0 kbp) which could not be unambigously placed due to the lack of spanning read pairs and a lack of similarity to any known *Acetobacteraecae* sequence in the NCBI non-redundant database^26^. The unplaced contigs may be part of the chromosome or pGHA01, or constitute separate plasmids. pGHA01 was determined to be a plasmid based on high sequence homology to the plasmid pGXY020 of *G. xylinus* NBRC 3288 (BLASTN against the nr database shows 99% identity with pGXY020 at 83% cover, E = 0.0).

To verify the accuracy of the genome sequence, we firstly designed primers based on genome sequence and amplified the *acsABCD* region by PCR. This yielded the expected product size (Supplementary Fig. S1 online), indicating that there are no discrepancies in the size of this region between genomic sequence and experiment. Secondly, we compared the genome sequence to regions of ATCC 53582 previously sequenced by other authors: *acsABCD* (GenBank: X54676.1)^16^,^19^, *cmcax* and *ccpAx* (GenBank: AB091058.1)^12^ and *bglxA* (GenBank: AB091059.1)^12^. We found that these sequences were a close match to their genome sequences: BLASTN of these sequences against the ATCC 53582 genome showed that in total only ten nucleotides differed out of 15,245 (4 SNPs, 6 indels) (see Supplementary Data S1 for full alignments). Evaluating sequence reads at these locations showed that these differences were not caused by sequencing errors in our genome sequence, as all reads covering the *acs1* operon contained these changes. We also evaluated whether read coverage was unusually high or low in this area, which may indicate sequencing biases or other issues. Although read coverage of the *acs1 operon* was slightly below average (RPKM = 360 vs 470 average of the genome), this difference was statistically not significant (p = 0.22, two-tailed t-test, comparing read coverage of all genes vs read coverage of genes in *acs1* operon), indicating that the differences were not caused by sequencing biases or errors. Of the six indels, five were found clumped close to the beginning of the *acsABCD* sequence previously reported by Saxena *et al.* (1990)^16^ (sequence X54676.1). These were likely sequencing errors in this sequence, as they conflict with both the sequence provided by Kawano *et al.* (2002)^12^ (sequence AB091058.1) as well as our genomic sequence. The 6^th^ indel, (a 1 bp deletion at the end of *acsA*, at genomic position 671834) had been previously confirmed by Saxena *et al.* (1994)^17^ as correct. Therefore, the genome sequence seems to differ in only 4 SNPs from previously published sequences, which are likely due to genuine differences – one in the *ccpAx* gene (a silent mutation), and three in *bglxA* gene causing V587A, D528N, R721W changes in the amino acid sequence. Whether these changes are functionally important is unknown.

Upon searching for genes related to cellulose synthesis, we found the *acs1* operon that had been previously described^12^,^16^,^17^, and which contains the full set of cellulose synthase genes (*acsAB1, acsC1* and *acsD1*), as well as *cmcax*, *ccpAx* upstream and *bglxA* downstream of the *acs* operon (Fig. 1). *cmcax* encodes for endo-β-1,4-glucanase^27^,^28^ and *bglxA* encodes for β-glucosidase^29^, both of which hydrolyse cellulose^27^,^28^,^29^. The function *ccpAx* is unknown^2^, however all three genes have been shown to be necessary for cellulose productivity^2^,^12^,^30^,^31^. In addition to the *bglxA* gene downstream of *acs1* operon, we found a second *bglxA* gene (*bglxA2*) at genomic positions 507976–510045. BglxA2 shares 31% amino acid sequence identity with BglxA1 (Supplementary Fig. S2), is distant from the *acs1* operon and is not flanked by any other obvious cellulose-related genes.

In addition to the *acs1* operon, we found two additional *acs* operons (*acs2, 3*), which differ in structure from the *acs1* operon. The *acs2* and *acs3* operons contain *acsAB* and *acsC* genes, but not *acsD* (the only copy of this seems to be in *acs1* operon). The *acs2* operon is located on the reverse strand, and contains *acsAB2* and *acsC2,* with two additional genes homologous to *bcsY* and *bcsX* in the middle of *acsAB2* and *acsC2* (Fig. 1A). *bcsY* is closely related to transacetylases and has been proposed to produce acetylated cellulose in *Acetobacter xylinum* JCM 7664^32^. *bcsX* may function in cellulose synthesis, but its function is not well known^32^. The *acs3* operon contains *acsAB3* and *acsC3* genes, without any other evident cellulose synthesis related genes (Fig. 1A). A phylogeny based on concatenated amino acid sequences of AcsAB and AcsC proteins suggests that *acs2* and *acs3* operons are more closely related to each other than the full *acs1* operon (Fig. 1B). *acs2* and *acs3* also do not contain *acsD*, *cmcax*, *ccpax* or *bglxA* genes, which suggests that these operons may have arisen as duplications of *acs1*, followed by divergence and loss of genes during evolution.

As activity of AcsAB is controlled by the allosteric activator c-di-GMP^4^, we also searched for genes associated with production and regulation of c-di-GMP. We found two Cdg operons (*cdg1* and *cdg2*) containing a diguanylate cyclase gene (*dgc1, 2*) followed by a c-di-GMP phosphodiesterase (*pdeA1, 2*), and finally, four standalone c-di-GMP phosphodiesterases (*pdeA3*–6) from the genome, which share 38–72% amino acid sequence identity (Supplementary Fig. S2). These genes together control the intracellular c-di-GMP levels, as diguanylate cyclases catalyse c-di-GMP synthesis while c-di-GMP phosphodiesterases degrade c-di-GMP^33^. This multiplicity of c-di-GMP regulatory genes has been noted in other bacteria^33^ and suggests multiple environmental signals are possibly integrated into the control over cellulose synthesis in ATCC 53582.

**Figure 2:**
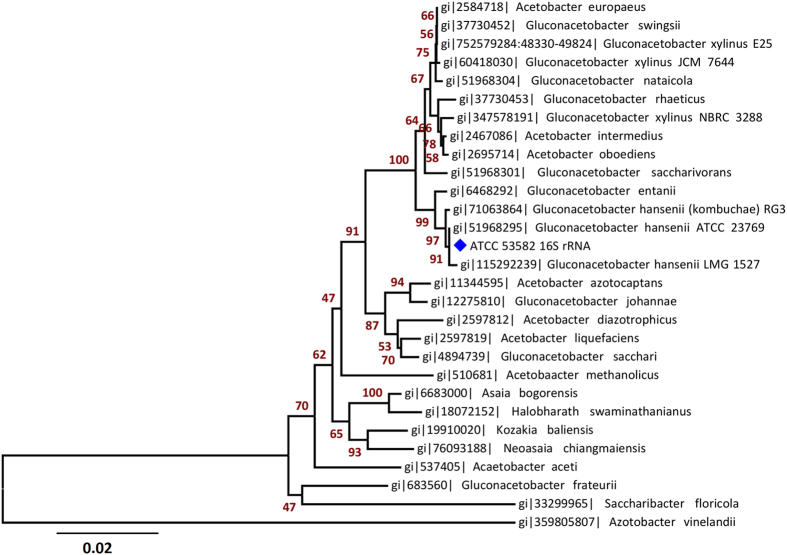
16s rRNA phylogeny of *G. hansenii* ATCC 53582 and *Acetobacteraceae* species. Phylogeny suggests the ATCC 53582 strain to be most closely related to *G. hansenii* ATCC 23769 and *G. hansenii* LMG 1527 (*G. hansenii* are more commonly used, but were recently reclassified as *K. hansenii* and are denoted in NCBI as such)^34^. The ATCC 53582 strain is denoted with a blue star. Nucleotide sequences were aligned, percent identity calculated using MUSCLE^54^ and the tree built using the Neighbour-Joining method. The tree is drawn to scale, with Bootstrap values from 1000 replicates shown next to the branches. All nucleotide positions containing gaps were removed from analysis. Analyses were conducted using MEGA6^55^.

To determine the phylogenetic relationship of ATCC 53582 to other *Acetobacteraceae*, we constructed a phylogeny using 16s rRNA from ATCC 53582 and other *Acetobacteraceae* (Fig. 2). The tree suggests ATCC 53582 to be closely related to *Gluconacetobacter hansenii* ATCC 23769, *Gluconacetobacter hansenii* LMG 1527 (previously *Gluconacetobacter xylinus*)^34^,^35^ and *Gluconacetobacter hansenii* RG3. This is also supported by the high sequence identity of 16s rRNA in these strains (99.1–99.7%) (Supplementary Fig. S3). Note also that while the ATCC 53582 and ATCC 23769 strains are denoted as *Gluconacetobacter xylinus* in many publications, *Acetobacter* taxonomy is undergoing constant revision, and *Gluconacetobacter xylinus* were classified as *Gluconacetobacter hansenii*^34^ and recently again as *Komagataeibacter hansenii*^35^.

### Transformation of *G. hansenii* ATCC 53582

**Figure 3:**
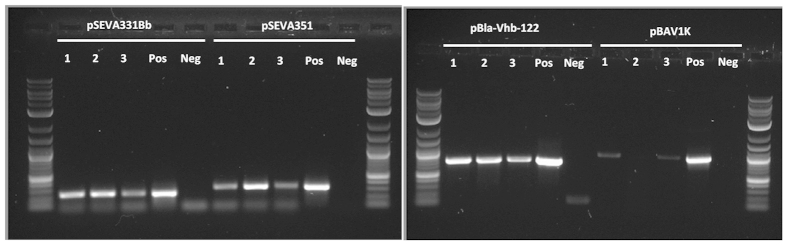
Colony PCR of *G. hansenii* ATCC 53582 transformed with pSEVA331Bb, pSEVA351, pBla-Vhb-122 and pBAV1K. 1, 2 and 3 – biological replicates of transformed ATCC 53582 used for colony PCR; Pos – Positive control (PCR with pure plasmid DNA), Neg – negative control (colony PCR of untransformed ATCC 53582); L – NEB Quick-Load Purple 2-log DNA ladder. ATCC 53582 were transformed with pSEVA311, pSEVA321, pSEVA331Bb, pSEVA341, pSEVA351, pBla-Vhb-122, pBAV1K, pSB1C3 and pBca1020. Of the used plasmids, colonies were present with pSEVA331Bb, pSEVA351, pBla-Vhb-122 and pBAV1K, and transformants were subsequently confirmed with colony PCR. Although failure to obtain colonies with other plasmids may be due to low transformation efficiencies, we were unable to obtain any transformants with these plasmids in repeat experiments, indicating incompatible origins of replication.

Various studies have aimed to increase cellulose production of *Acetobacter* strains^2^,^21^,^31^,^36^. ATCC 53582 is naturally a high-producing strain^12^,^13^, and is therefore potentially a good platform for further optimization via genetic engineering. Hall *et al.* (1992)^37^ had shown that *G. hansenii* can be transformed via electroporation^37^, and electroporation was similarly used by Chien *et al.* (2006)^21^ and Setyawati *et al.* (2007)^20^. However, ATCC 53582 has been reported to be difficult to transform – while Saxena *et al.* (1994)^17^ could transform other *Gluconacetobacter* strains, they reported being unable to transform the ATCC 53582 strain^17^. We used electroporation as described by Hall *et al.* (1992)^37^, Chien *et al.* (2006)^21^, Dower *et al.* (1998)^38^ and Deng *et al.* (2013)^39^. These protocols were assessed with the plasmid pSEVA331Bb, which is capable of replication in the closely related *Komagataeibacter rhaeticus* iGEM strain^40^. In agreement with past observations by Saxena *et al.* (1994)^17^ we were unable to obtain any successful transformants with *G. hansenii* ATCC 53582 using these previously described transformation protocols. In an effort to determine whether this was caused by suboptimal transformation conditions, we systematically altered parameters for the production of electrocompetent cells and electroporation from the protocol described by Hall *et al.* (1992). We were able to achieve transformants only when using a 3 kV pulse and a long post-transformation incubation time (see Materials and Methods and Supplementary Methods online for details), however the transformation efficiency remained low (approximately 10^2^ CFU/μg DNA) and no transformants could be obtained with three pSEVA331Bb-based plasmids containing fluorescent reporters (plasmids pSEVA331Bb-P_J23100_-mRFP1, pSEVA331Bb-P_J23101_-mRFP1 and pSEVA331Bb-P_J23104_-mRFP1, where mRFP1 is expressed behind different constitutive promoters P_J23100_ P_J23101_ and P_J23104_). Nevertheless, the reproducibility of the protocol was verified, and transformation and propagation of the pSEVA331Bb backbone was confirmed despite low efficiencies (Fig. 3, Supplementary Fig. S4).

Using this protocol, we tested 8 additional plasmids with different replication origins and selectable markers for their ability to propagate in ATCC 53582: pSEVA311, pSEVA321, pSEVA341, pSEVA351; pBla-Vhb-122, pBAV1K, pSB1C3 and pBca1020 (see Table S1 for details). From the tested plasmids, we obtained successful transformants with pSEVA351, pBla-Vhb-122 and pBAV1K in addition to pSEVA331Bb (Fig. 3). Although transformation efficiencies were low for all plasmids, we generally observed higher efficiencies with pSEVA331Bb and pSEVA351, which are likely the best options as backbones. For pSEVA321, pSEVA341, pSB1C3 and pBca1020, were unable to obtain any transformants even in repeated experiments.

As genomic restriction enzymes can reduce transformation efficiencies, we searched the ATCC 53582 genome for genes encoding restriction enzymes and identified two systems - a predicted restriction enzyme from the Mrr family (genomic positions 2827888–2829030), and a locus containing a homologue of PstI and its likely methyltransferase (genomic positions 2399354–2401806). Mrr is unlikely to cause the low transformation efficiencies observed, as it targets methylated DNA in certain sequence contexts^41^ and has been reported not to restrict transformation of unmethylated plasmids^42^. On the other hand, the recognition sequence of PstI (CTGCAG) was present in single-copy in all of our tested plasmids, raising the possibility that a native PstI may be a cause of low transformation efficiencies. To test this, we mutated the PstI cleavage site (CTGCAG -> CTGCAC) in pSEVA331Bb (pSEVA331Bb-PstI^-^) and in three plasmids containing fluorescent reporters (pSEVA331Bb-P_J23100_-mRFP1-PstI^-^, pSEVA331Bb-P_J23101_-mRFP1-PstI^-^ and pSEVA331Bb-P_J23104_-mRFP1-PstI^-^). While transformants could be obtained with pSEVA331Bb-PstI^-^, the observed efficiencies were no higher compared to pSEVA331Bb, and once again no transformants were obtained with plasmids harbouring mRFP1 expression cassettes. Thus this suggests that low transformation efficiencies are likely caused by different mechanisms.

## Discussion

Our results suggest that pSEVA331Bb, pSEVA351, pBla-Vhb-122 and pBAV1K can replicate in *G. hansenii* ATCC 53582 (Fig. 3), and may be used as vectors for genetic engineering. For pSEVA311, pSEVA321, pSEVA341, pSB1C3 and pBca1020, we were unable to obtain any transformants despite repeated attempts. Although this may have been caused by low transformation efficiencies, we saw similar results (with the exception of the very low-copy number pSEVA321) in the closely related *K. rhaeticus* iGEM, where pSEVA311, pSEVA341, pSB1C3 and pBca1020 similarly showed no replication^40^. The lack of replication is unlikely to have been caused by differences in the numbers of PstI cleavage sites or different selectable markers, as all plasmids contained one PstI site and chloramphenicol was used as the selectable marker in most cases (Supplementary Table S1). This strongly indicates that the plasmids unable to replicate in *G. hansenii* ATCC 53582 (pSEVA311, pSEVA341, pSB1C3 and pBca1020) failed to do so due to non-compatibility of their replication origins with this species.

Despite the low transformation efficiencies, plasmid propagation and antibiotic resistance indicates that plasmid-based protein expression is clearly occurring. However, addition of mRFP1 expression cassettes to pSEVA331Bb decreased efficiencies to levels where no transformed colonies could be obtained, possibly due to a combination of increased plasmid size and increased metabolic burden on cells. While the presence of a genomic *pstI* homologue offered a possible explanation for low efficiencies, experimental evidence indicates that this is not the main cause, as removal of PstI restriction sites from plasmids did not result in increased number of transformed colonies. The mechanism for low transformation efficiencies in ATCC 53582 therefore remains an open question. While the protocol and plasmid backbones reported here offer a starting point for genetic engineering of ATCC 53582, it is clear that ATCC 53582 remains difficult to engineer, and in its current state is a suboptimal host for genetic engineering. As has been the case with many other model organisms including *E. coli*, increasing transformation rates and obtaining robust heterologous gene expression will likely require discovery or generation of alternative strains that have improved properties that enable genetic engineering.

In addition to PstI and Mrr restriction systems, the genome sequence revealed several other interesting features. We identified two *Cdg* operons containing a diguanylate cyclase and a c-di-GMP phosphodiesterase, and four additional c-di-GMP phosphodiesterases from the genome. Diguanylate cyclases and c-di-GMP phosphodiesterases control c-di-GMP levels by synthesising and degrading c-di-GMP respectively^33^. In a closely related *Acetobacter* species, 3 *Cdg* operons have been identified, and it was reported that disruption of these operons influenced cellulose productivity to different extents^43^, suggesting that these operons have specialized roles in c-di-GMP regulation. Multiplicity of c-di-GMP regulatory genes allows for multiple signals to be incorporated into c-di-GMP control, and has also been found in many other bacteria^33^, suggesting that control over cellulose synthesis in ATCC 53582 may similarly be complex and affected by multiple environmental signals.

Previously, a single *acs* operon had been identified in *G. hansenii* ATCC 53582^17^, and a second one had been characterized in related strains^32^,^44^. For other *Acetobacteraceae* species for which the genome has been sequenced, the authors reported one *acs* operon in the cellulose non-producing *G. xylinus* NBRC3288^25^ and two *acs* operons for *G. xylinus* E25^23^. For ATCC 53582, genome sequence shows presence of three operons (Fig. 1), which may explain the high cellulose productivity observed. However, it is important to note that three *acs* operons were similarly noted in *G. hansenii* ATCC 23769^24^, despite having 5 times lower cellulose productivity than ATCC 53582 on glucose^12^, and that strains with *acsA2* knockouts did not suffer from decreased cellulose productivity^44^. Therefore, although having multiple copies of cellulose synthase operons may allow for increased cellulose synthesis in ATCC 53582, it is likely that the copy number of *acs* operons alone is not the main cause of high cellulose productivity, but that differences in gene expression levels, regulation of AcsAB activity by c-di-GMP, or glucose metabolism may be more important.

## Materials and Methods

### Culturing of *Gluconacetobacter hansenii* ATCC 53582

*Gluconacetobacter hansenii* ATCC 53582 was purchased from ATCC (cat. number 53582, ATCC – Middlesex, UK) and streaked on HS-agarose containing 2% (w/v) glucose. Single colonies were then picked and grown statically in liquid HS at 30 °C for 6 days. Culture was then incubated with 0.2% (v/v) cellulase (*T. reesei* cellulase, cat. no. C2730 – Sigma, St. Louis USA) at 230 rpm shaking, 30 °C for 24 hours to digest cellulose, and resulting culture stored in 25% (v/v) glycerol, −80 °C as glycerol stocks. For all subsequent experiments, seed cultures were prepared from glycerol stocks.

### Genome sequencing, assembly and bioinformatics

For genomic DNA (gDNA) extraction, liquid HS was inoculated from glycerol stocks, grown for 7 days at 30 °C, standing, and cellulose digested via addition of 0.2% (v/v) cellulase and incubation at 30 °C, 230 rpm for 24 hours. 5 mL culture was centrifuged in 50 mL Corning tubes (cat. no. 430290 – Corning Costar, New York USA), at 3200 g for 10 minutes at 4 °C, supernatant discarded, and cells re-suspended in 5 mL cold HS. This was repeated twice in total to remove cellulase and other contaminants present in the medium. gDNA was then extracted from the resulting culture using DNeasy Blood and Tissue kit (cat. no. 69504–Qiagen; Venlo, Netherlands) according to manufacturer’s protocol. To remove contaminants, DNA was purified using Zymo Clean and Concentrator kit (cat. no. D4003–Zymo Research, California, USA) according to manufacturer’s instructions. DNA was then further purified by dialyzing 30 μL of DNA on a 0.025 μm filter (cat. no. VSWP02500–Merck Millipore, Massachusetts, USA) for 1 hour. gDNA sequencing library was then prepared with Nextera DNA Library Preparation Kit (cat. no. FC-121-1031; Illumina–San Diego, USA) according to manufacturer’s protocol. Library was sequenced on an Illumina MiSeq (Illumina) using 250 bp, paired-end reads, to a coverage of approximately 800x.

Before assembly, reads were quality controlled using FastQC^45^. Reads were downsampled to approximately 100x coverage and assembled using the BugBuilder pipeline^22^, using Sickle^46^ for trimming reads (with read areas of quality score below 20 trimmed), Spades^47^ for assembly (with full read set) and SIS^48^ for scaffolding, with *G. xylinus* NBRC 3288^25^ as a reference genome for scaffolding. This was followed by manual correction and verification of scaffolds, using the NBRC 3288 genome as a reference. Assembled genome was then subjected to quality control using Quast^49^, mis-assemblies manually corrected, and edited genome checked again using Quast. Gapfiller^50^ was then used to fill gaps within scaffolds. Origin of replication was located using DoriC^51^ and scaffolds manually reorganized to position the origin at the beginning of the genome. The genome was annotated using Prokka^52^, all cellulose synthesis and c-di-GMP production related genes, as well as restriction enzymes were manually checked by BLASTP and BLASTN against the non-redundant database^26^ and re-annotated as necessary. Genes were also searched from the genome using BLAST+^53^ by converting the finished assembly and raw reads to BLAST databases and subjecting them to BLASTN, TBLASTN or TBLASTX searches with genes of interest. 16s rRNA phylogeny was created by generating a multiple sequence alignment with MUSCLE^54^ and a Neighbour-Joining tree using MEGA6 package^55^ at default settings. Reads were mapped onto the genome using BWA and genome was visualized using Circleator^56^.

The presence of genomic *pstI* in *G. hansenii* ATCC 53582, *K. rhaeticus* iGEM, *G. hansenii* ATCC 23769 and *G. xylinus* E25 was searched using *pstI* reference sequence from *Clostridium perfringens* (NCBI ID: 18990735) and BLASTN and TBLASTX at default settings, with the *G. hansenii* ATCC 53582 genome reported here, the unpublished *Komagataeibacter rhaeticus* iGEM genome (Registry of Standard Biological Parts ID: BBa_K1321306)^40^ and the published *G. hansenii* ATCC 23769^24^ and *G. xylinus* E25^23^ genomes used respectively. Unlike for ATCC 53582 (E-value = 10^–73^), no significant matches were found for *K. rhaeticus* iGEM, *G. hansenii* ATCC 23769 and *G. xylinus* E25 (E = 4.4, 0.27 and no hits respectively) for *pstI*, indicating that it is not present in these species.

### Production of electrocompetent cells and transformation

For production of electrocompetent cells, firstly a seed culture was prepared by inoculating 5 mL of HS-0.2% (v/v) cellulase in 50 mL Corning tubes from glycerol stocks and incubating at 30 °C, 230 rpm shaking, 45° tube angle for 24–72 hours, or until OD_600_ > 0.7. Then, 10 mL of HS + cellulase medium was added into each of 8 of 50 mL Corning tubes, and seed culture added to a final OD_600_ of 0.04. Tubes were then incubated at 30 °C, 230 rpm shaking, 45° tube angle until OD_600_ reached 0.4 … 0.7. Cells were then centrifuged at 3200 g at 4 °C for 12 minutes, supernatant removed, and cell pellicles resuspended in 10 mL 4 °C HEPES for each tube. Cells were similarly washed once more, pooled, and re-suspended in a total of 6 mL 4 °C 15% glycerol. 100 μL aliquots of this were then stored at −80 °C and used for electroporation.

ATCC 53582 was transformed via electroporation. 2–6 μL of pure, dialyzed DNA was added to 100 μL of electrocompetent cells on ice and incubated for 5–15 minutes. The cell-DNA mixture was electroporated using 0.1 cm Gene Pulser Electrocuvettes (cat. no. 1652089, BioRad, Hertfordshire UK) and BioRad Micropulser (BioRad) set at 3 kV, 5–8 ms. Cells were then re-suspended and grown in 800 μL HS-0.1% (v/v) cellulase medium for 16 hours, plated on HS-agar plates containing 34 μg/mL chloramphenicol (for pSEVA311-351 and pSB1C3), 50 μg/mL ampicillin (for pBca1020) or 100 μg/mL of kanamycin (for pBAV1K and pBla-Vhb-122), and incubated inverted for 3–6 days at 30 °C. Any appearing colonies were tested with colony PCR (see Supplementary Table S2 for primers) or grown for testing with subsequent plasmid DNA extraction. See Supplementary Methods online for protocols of electrocompetent cell preparation and transformation.

### Molecular biology and cloning

pSEVA311, pSEVA321, pSEVA331, pSEVA341, pSEVA351 were received from the SEVA collection^57^, pBAV1K^58^ was purchased from Addgene (cat. no. 26702) – Addgene, Massachusetts, USA), pBla-Vhb-122 was kindly sent by Chien *et al.* (2006)^21^, and pSB1C3 and pBca1020 from the Registry of Standard Biological Parts^59^. Note that although pBAV1K contains a GFP gene, there is no functional expression of GFP from this plasmid, as our sequencing upon receipt of this plasmid revealed a deletion in the GFP promoter region (see Supplementary Data S2 for sequence). pSEVA331Bb was engineered from pSEVA331 by replacing the native multiple cloning site sequence with a BioBrick cloning sequence containing the prefix and suffix for compatibility with the BioBrick standard^60^ (see Supplementary Data S3 for sequence).

pSEVA331Bb-P_J23100_-mRFP1, pSEVA331Bb-P_J23101_-mRFP1 and pSEVA331Bb-P_J23104_-mRFP1 were engineered from pSEVA331Bb by restriction cloning mRFP1 expression cassettes (P_J23100_-mRFP1, P_J23101_-mRFP1 and P_J23104_-mRFP1) from plasmids BBa_J23100, BBa_J23101 and BBa_J23104 available from the Registry of Standard Biological Parts into pSEVA331Bb (see Supplementary Data S4–S6 for plasmid maps, all sequences are in GenBank format). To remove PstI cleavage site while maintaining plasmid size and GC content, the PstI recognition sequence was mutagenized from CTGCAG to CTGCAC using inverse PCR with mutagenic primers (plasmids pSEVA331Bb-P_J23100_-mRFP1-PstI^-^, pSEVA331Bb-P_J23101_-mRFP1-PstI^-^ and pSEVA331Bb-P_J23104_-mRFP1-PstI^-^). Successful mutagenesis was confirmed using test restriction digests.

For colony PCR, transformed colonies were screened using GoTaq Green (cat. no. M5122 – Promega, Madison USA), in 20 μl reaction volume. For long-range PCR of the *acs* operon, Q5 HF polymerase (cat. no. M0491S – NEB, Massachussets, USA) was used in 50 μL reactions, with or without 10 μL GC enhancer 1 μL template DNA according to manufacturer’s instructions. Thermocycler programs for both colony and long-range PCR are listed in Supplementary Table S3. For plasmid DNA extraction, single colonies were picked from plates after transformation with pSEVA331Bb and inoculated into 5 mL of HS media with 2% (w/v) glucose and 0.2% (v/v) cellulase in a 50 mL tube and incubated at 30 °C with shaking for 48 hours. Plasmid DNA was then extracted from the cultures using a QIAprep spin miniprep kit (Qiagen N.V.) according to manufacturer’s instructions. The prepared plasmids were digested with NcoI restriction enzyme (NEB Inc.) and analysed on agarose gels. The ladder used was NEB Quick-Load Purple 2-log DNA ladder (cat. no. N0550S – NEB) for all tests.

## Additional Information

**Accession codes**: Genome assembly and all associated sequence data have been submitted to the ENA as project ID PRJEB10804. 

**How to cite this article**: Florea, M. *et al.* Genome sequence and plasmid transformation of the model high-yield bacterial cellulose producer *Gluconacetobacter hansenii* ATCC 53582. *Sci. Rep.* **6**, 23635; doi: 10.1038/srep23635 (2016).

## Supplementary Material

Supplementary Data S1

Supplementary Data S2

Supplementary Data S3

Supplementary Data S4

Supplementary Data S5

Supplementary Data S6

Supplementary Information

## References

[b1] Williams W. S. & Cannon R. E. Alternative Environmental Roles for Cellulose Produced by *Acetobacter xylinum*. Appl. Environ. Microbiol. 55, 2448–52 (1989). 16348023 10.1128/aem.55.10.2448-2452.1989 PMC203103

[b2] Lee K.-Y., Buldum G., Mantalaris A. & Bismarck A. More than meets the eye in bacterial cellulose: biosynthesis, bioprocessing, and applications in advanced fiber composites. Macromol. Biosci. 14, 10–32 (2014). 23897676 10.1002/mabi.201300298

[b3] Wong H. C. *et al.* Genetic organization of the cellulose synthase operon in *Acetobacter xylinum*. Proc. Natl. Acad. Sci. USA 87, 8130–4 (1990). 2146681 10.1073/pnas.87.20.8130 PMC54906

[b4] Ross P. *et al.* The cyclic diguanylic acid regulatory system of cellulose synthesis in *Acetobacter xylinum*. Chemical synthesis and biological activity of cyclic nucleotide dimer, trimer, and phosphothioate derivatives. J. Biol. Chem. 265, 18933–43 (1990). 2172238

[b5] Hsieh Y. C., Yano H., Nogi M. & Eichhorn S. J. An estimation of the Young’s modulus of bacterial cellulose filaments. Cellulose 15, 507–513 (2008).

[b6] Sun S. *et al.* Comparison of the mechanical properties of cellulose and starch films. Biomacromolecules 11, 126–32 (2010). 19908856 10.1021/bm900981t

[b7] Klemm D., Schumann D., Udhardt U. & Marsch S. Bacterial synthesized cellulose — artificial blood vessels for microsurgery. Prog. Polym. Sci. 26, 1561–1603 (2001).

[b8] Yadav V. *et al.* Novel *in vivo*-degradable cellulose-chitin copolymer from metabolically engineered *Gluconacetobacter xylinus*. Appl. Environ. Microbiol. 76, 6257–65 (2010). 20656868 10.1128/AEM.00698-10 PMC2937499

[b9] Ummartyotin S., Juntaro J., Sain M. & Manuspiya H. Development of transparent bacterial cellulose nanocomposite film as substrate for flexible organic light emitting diode (OLED) display. Ind. Crops Prod. 35, 92–97 (2012).

[b10] Hu W. *et al.* Highly stable and sensitive humidity sensors based on quartz crystal microbalance coated with bacterial cellulose membrane. Sensors Actuators B Chem. 159, 301–306 (2011).

[b11] Hu W., Chen S., Yang J., Li Z. & Wang H. Functionalized bacterial cellulose derivatives and nanocomposites. Carbohydr. Polym. 101, 1043–60 (2014). 24299873 10.1016/j.carbpol.2013.09.102

[b12] Kawano S. *et al.* Cloning of cellulose synthesis related genes from *Acetobacter xylinum* ATCC23769 and ATCC53582: comparison of cellulose synthetic ability between strains. DNA Res. 9, 149–56 (2002). 12465714 10.1093/dnares/9.5.149

[b13] Fang L. & Catchmark J. M. Characterization of cellulose and other exopolysaccharides produced from *Gluconacetobacter* strains. Carbohydr. Polym. 115, 663–9 (2015). 25439946 10.1016/j.carbpol.2014.09.028

[b14] Bureau T. E. & Brown R. M. *In vitro* synthesis of cellulose II from a cytoplasmic membrane fraction of *Acetobacter xylinum*. Proc. Natl. Acad. Sci. USA 84, 6985–9 (1987). 16593877 10.1073/pnas.84.20.6985 PMC299213

[b15] Lin F. C., Brown R. M., Drake R. R. & Haley B. E. Identification of the uridine 5′-diphosphoglucose (UDP-Glc) binding subunit of cellulose synthase in *Acetobacter xylinum* using the photoaffinity probe 5-azido-UDP-Glc. J. Biol. Chem. 265, 4782–4 (1990). 2138620

[b16] Saxena I. M., Lin F. C. & Brown R. M. Cloning and sequencing of the cellulose synthase catalytic subunit gene of *Acetobacter xylinum*. Plant Mol. Biol. 15, 673–83 (1990). 2151718 10.1007/BF00016118

[b17] Saxena I. M., Kudlicka K., Okuda K. & Brown R. M. Characterization of genes in the cellulose-synthesizing operon (acs operon) of *Acetobacter xylinum*: implications for cellulose crystallization. J. Bacteriol. 176, 5735–52 (1994). 8083166 10.1128/jb.176.18.5735-5752.1994 PMC196778

[b18] Kato N. *et al.* Viability and cellulose synthesizing ability of *Gluconacetobacter xylinus* cells under high-hydrostatic pressure. Extremophiles 11, 693–8 (2007). 17643184 10.1007/s00792-007-0085-y

[b19] Saxena I. M., Lin F. C. & Brown R. M. Identification of a new gene in an operon for cellulose biosynthesis in *Acetobacter xylinum*. Plant Mol. Biol. 16, 947–54 (1991). 1830823 10.1007/BF00016067

[b20] Setyawati M. I., Chien L.-J. & Lee C.-K. Expressing Vitreoscilla hemoglobin in statically cultured *Acetobacter xylinum* with reduced O(2) tension maximizes bacterial cellulose pellicle production. J. Biotechnol. 132, 38–43 (2007). 17868946 10.1016/j.jbiotec.2007.08.012

[b21] Chien L.-J., Chen H.-T., Yang P.-F. & Lee C.-K. Enhancement of cellulose pellicle production by constitutively expressing vitreoscilla hemoglobin in *Acetobacter xylinum*. Biotechnol. Prog. 22, 1598–603 (2006). 17137307 10.1021/bp060157g

[b22] Abbott J. (2015). BugBuilder: Microbial Genome Assembly Pipeline. Imperial College London, London, United Kingdom. URL: http://www3.imperial.ac.uk/bioinfsupport/resources/software/bugbuilder (Accessed: 7 September 2015)

[b23] Kubiak K. *et al.* Complete genome sequence of *Gluconacetobacter xylinus* E25 strain–valuable and effective producer of bacterial nanocellulose. J. Biotechnol. 176, 18–9 (2014). 24556328 10.1016/j.jbiotec.2014.02.006

[b24] Iyer P. R., Geib S. M., Catchmark J., Kao T. & Tien M. Genome sequence of a cellulose-producing bacterium, *Gluconacetobacter hansenii* ATCC 23769. J. Bacteriol. 192, 4256–7 (2010). 20543071 10.1128/JB.00588-10 PMC2916434

[b25] Ogino H. *et al.* Complete genome sequence of NBRC 3288, a unique cellulose-nonproducing strain of *Gluconacetobacter xylinus* isolated from vinegar. J. Bacteriol. 193, 6997–8 (2011). 22123756 10.1128/JB.06158-11 PMC3232855

[b26] Pruitt K. D., Tatusova T. & Maglott D. R. NCBI Reference Sequence (RefSeq): a curated non-redundant sequence database of genomes, transcripts and proteins. Nucleic Acids Res. 33, D501–4 (2005). 15608248 10.1093/nar/gki025 PMC539979

[b27] Standal R. *et al.* A new gene required for cellulose production and a gene encoding cellulolytic activity in *Acetobacter xylinum* are colocalized with the bcs operon. J. Bacteriol. 176, 665–72 (1994). 8300521 10.1128/jb.176.3.665-672.1994 PMC205103

[b28] Koo H. M., Song S. H., Pyun Y. R. & Kim Y. S. Evidence that a beta-1,4-endoglucanase secreted by *Acetobacter xylinum* plays an essential role for the formation of cellulose fiber. Biosci. Biotechnol. Biochem. 62, 2257–9 (1998). 9972249 10.1271/bbb.62.2257

[b29] Tonouchi N. *et al.* A beta-glucosidase gene downstream of the cellulose synthase operon in cellulose-producing *Acetobacter*. Biosci. Biotechnol. Biochem. 61, 1789–90 (1997). 9362130 10.1271/bbb.61.1789

[b30] Kawano S. *et al.* Effects of endogenous endo-beta-1,4-glucanase on cellulose biosynthesis in *Acetobacter xylinum* ATCC23769. J. Biosci. Bioeng. 94, 275–81 (2002). 16233303 10.1263/jbb.94.275

[b31] Tonouchi N. *et al.* Addition of a Small Amount of an Endoglucanase Enhances Cellulose Production by *Acetobacter xylinum*. Biosci. Biotechnol. Biochem. 59, 805–808 (1995).

[b32] Umeda Y. *et al.* Cloning of cellulose synthase genes from *Acetobacter xylinum* JCM 7664: implication of a novel set of cellulose synthase genes. DNA Res. 6, 109–15 (1999). 10382968 10.1093/dnares/6.2.109

[b33] Hengge R. Principles of c-di-GMP signalling in bacteria. Nat. Rev. Microbiol. 7, 263–73 (2009). 19287449 10.1038/nrmicro2109

[b34] Lisdiyanti P., Navarro R. R., Uchimura T. & Komagata K. Reclassification of *Gluconacetobacter hansenii* strains and proposals of *Gluconacetobacter saccharivorans sp. nov.* and *Gluconacetobacter nataicola sp. nov*. Int. J. Syst. Evol. Microbiol. 56, 2101–11 (2006). 16957106 10.1099/ijs.0.63252-0

[b35] Yamada Y. *et al.* Description of *Komagataeibacter gen. nov.*, with proposals of new combinations (*Acetobacteraceae*). *J*. Gen. Appl. Microbiol. 58, 397–404 (2012). 10.2323/jgam.58.397 23149685

[b36] Son H. J., Heo M. S., Kim Y. G. & Lee S. J. Optimization of fermentation conditions for the production of bacterial cellulose by a newly isolated *Acetobacter* sp. A9 in shaking cultures. Biotechnol. Appl. Biochem. 33, 1–5 (2001). 11171030 10.1042/ba20000065

[b37] Hall P. E., Anderson S. M., Johnston D. M. & Cannon R. E. Transformation of *Acetobacter xylinum* with plasmid DNA by electroporation. Plasmid 28, 194–200 (1992). 1461938 10.1016/0147-619x(92)90051-b

[b38] Dower W. J., Miller J. F. & Ragsdale C. W. High efficiency transformation of *E. coli* by high voltage electroporation. Nucleic Acids Res. 16, 6127–6145 (1988). 3041370 10.1093/nar/16.13.6127 PMC336852

[b39] Deng Y., Nagachar N., Xiao C., Tien M. & Kao T. Identification and characterization of non-cellulose-producing mutants of *Gluconacetobacter hansenii* generated by Tn5 transposon mutagenesis. J. Bacteriol. 195, 5072–83 (2013). 24013627 10.1128/JB.00767-13 PMC3811599

[b40] Imperial iGEM 2014 team. Registry of Standard Biological Parts. *Komagataeibacter rhaeticus* iGEM. (2015). Available at: http://parts.igem.org/Part:BBa_K1321306 (Accessed: 21 October 2015)

[b41] Loenen W. A. M. & Raleigh E. A. The other face of restriction: modification-dependent enzymes. Nucleic Acids Res. 42, 56–69 (2014). 23990325 10.1093/nar/gkt747 PMC3874153

[b42] Grant S. G., Jessee J., Bloom F. R. & Hanahan D. Differential plasmid rescue from transgenic mouse DNAs into *Escherichia coli* methylation-restriction mutants. Proc. Natl. Acad. Sci. USA 87, 4645–9 (1990). 2162051 10.1073/pnas.87.12.4645 PMC54173

[b43] Tal R. *et al.* Three cdg Operons Control Cellular Turnover of Cyclic Di-GMP in *Acetobacter xylinum*: Genetic Organization and Occurrence of Conserved Domains in Isoenzymes. J. Bacteriol. 180, 4416–4425 (1998). 9721278 10.1128/jb.180.17.4416-4425.1998 PMC107450

[b44] Saxena I. M. & Brown R. M. Identification of a second cellulose synthase gene (acsAII) in *Acetobacter xylinum*. J. Bacteriol. 177, 5276–83 (1995). 7665515 10.1128/jb.177.18.5276-5283.1995 PMC177319

[b45] Babraham Bioinformatics (2015). FastQC - A quality control tool for high throughput sequence data. Babraham Institute, Cambridge, United Kingdom. URL: http://www.bioinformatics.babraham.ac.uk/projects/fastqc/ (Accessed: 31 August 2015)

[b46] UC Davis Bioinformatics Core (2015). Sickle: A windowed adaptive trimming tool for FASTQ files using quality. UC Davis, California, United States of America. URL: http://bioinformatics.ucdavis.edu/software/ (Accessed: 7 September 2015)

[b47] Bankevich A. *et al.* SPAdes: a new genome assembly algorithm and its applications to single-cell sequencing. J. Comput. Biol. 19, 455–77 (2012). 22506599 10.1089/cmb.2012.0021 PMC3342519

[b48] Dias Z., Dias U. & Setubal J. C. SIS: a program to generate draft genome sequence scaffolds for prokaryotes. BMC Bioinformatics 13, 96 (2012). 22583530 10.1186/1471-2105-13-96 PMC3674793

[b49] Gurevich A., Saveliev V., Vyahhi N. & Tesler G. QUAST: quality assessment tool for genome assemblies. Bioinformatics 29, 1072–5 (2013). 23422339 10.1093/bioinformatics/btt086 PMC3624806

[b50] Boetzer M. & Pirovano W. Toward almost closed genomes with GapFiller. Genome Biol. 13, R56 (2012). 22731987 10.1186/gb-2012-13-6-r56 PMC3446322

[b51] Gao F. & Zhang C.-T. DoriC: a database of oriC regions in bacterial genomes. Bioinformatics 23, 1866–7 (2007). 17496319 10.1093/bioinformatics/btm255

[b52] Seemann T. Prokka: rapid prokaryotic genome annotation. Bioinformatics 30, 2068–9 (2014). 24642063 10.1093/bioinformatics/btu153

[b53] Camacho C. *et al.* BLAST+: architecture and applications. BMC Bioinformatics 10, 421 (2009). 20003500 10.1186/1471-2105-10-421 PMC2803857

[b54] Edgar R. C. MUSCLE: multiple sequence alignment with high accuracy and high throughput. Nucleic Acids Res. 32, 1792–7 (2004). 15034147 10.1093/nar/gkh340 PMC390337

[b55] Tamura K., Stecher G., Peterson D., Filipski A. & Kumar S. MEGA6: Molecular Evolutionary Genetics Analysis version 6.0. Mol. Biol. Evol. 30, 2725–9 (2013). 24132122 10.1093/molbev/mst197 PMC3840312

[b56] Crabtree J. *et al.* Circleator: flexible circular visualization of genome-associated data with BioPerl and SVG. Bioinformatics 30, 3125–7 (2014). 25075113 10.1093/bioinformatics/btu505 PMC4201160

[b57] Durante-Rodríguez G., de Lorenzo V. & Martínez-García E. The Standard European Vector Architecture (SEVA) plasmid toolkit. Methods Mol. Biol. 1149, 469–78 (2014). 24818926 10.1007/978-1-4939-0473-0_36

[b58] Bryksin A. V. & Matsumura I. Rational design of a plasmid origin that replicates efficiently in both gram-positive and gram-negative bacteria. Plos One 5, e13244 (2010). 20949038 10.1371/journal.pone.0013244 PMC2951906

[b59] iGEM Foundation. Registry of Standard Biological Parts. (2015). Available at: http://parts.igem.org/Main_Page (Accessed: 11 September 2015)

[b60] Shetty R. P., Endy D. & Knight T. F. Engineering BioBrick vectors from BioBrick parts. J. Biol. Eng. 2, 5 (2008). 18410688 10.1186/1754-1611-2-5 PMC2373286

