# Supplementary material for: Genome sequence and plasmid transformation of the model high-yield bacterial cellulose producer Gluconacetobacter hansenii ATCC 53582
Source: Sci Rep. 2016 Mar 24;6:23635. doi: 10.1038/srep23635 (PMC4806288; doi:10.1038/srep23635)
Supplement: Supplementary Information [file srep23635-s7.doc]

**Genome sequence and plasmid transformation of the model high-yield bacterial cellulose producer *Gluconacetobacter hansenii* ATCC 53582**

**Michael Florea^1, 2^, Benjamin Reeve^2,5^, James Abbott^3,4^, Paul S Freemont^2,6^, Tom Ellis^2,5^***

**1** Department of Life Sciences, Imperial College London, London, UK; **2** Centre for Synthetic Biology and Innovation, Imperial College London, London, UK; **3** Bioinformatics Support Service, Department of Surgery and Cancer, Imperial College London, London, UK; **4** Centre for Integrative Systems Biology and Bioinformatics, Imperial College London, London, UK; **5** Department of Bioengineering, Imperial College London, London, UK; **6** Department of Medicine, Imperial College London, London, UK;.

*****Corresponding author: [t.ellis@imperial.ac.uk](mailto:t.ellis@imperial.ac.uk) (TE)

# **Supplementary Figures**


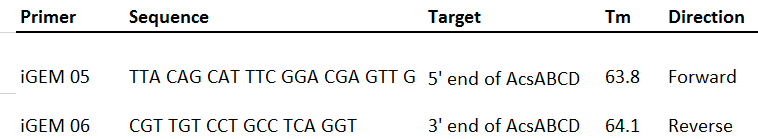

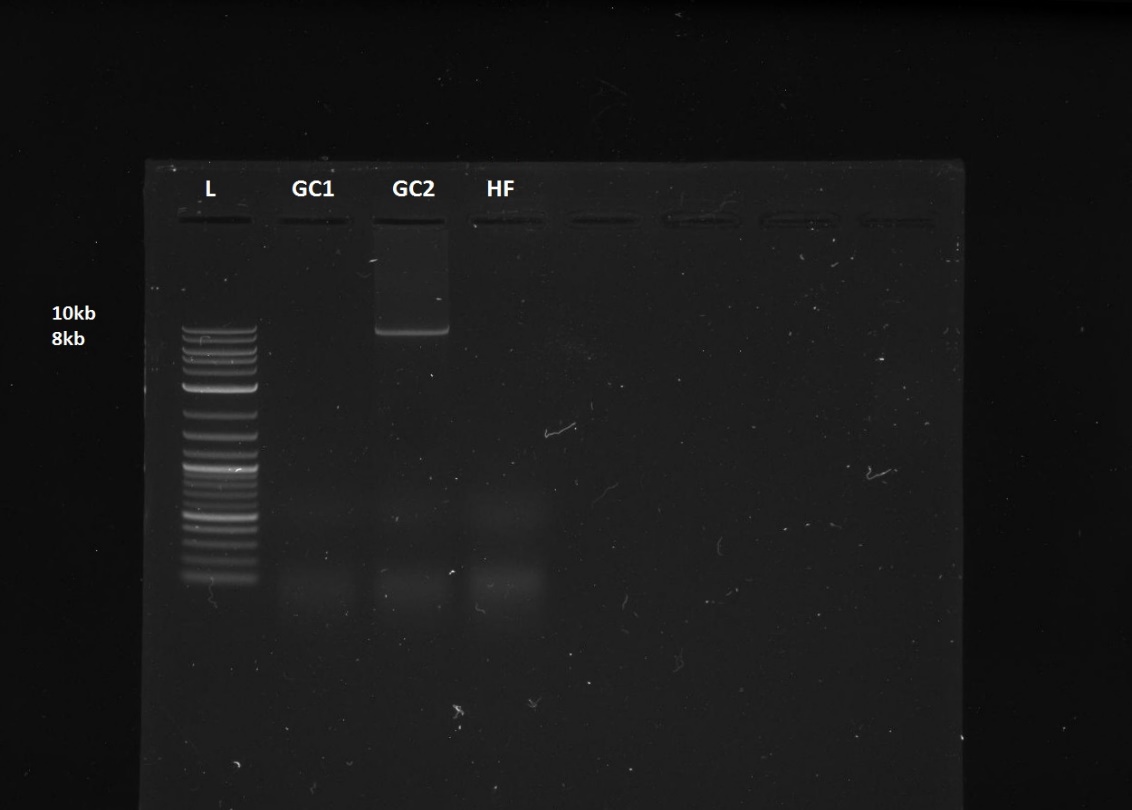


**Supplementary Figure S1. (A) Primers and (B) PCR of *acsABCD* genes from *acs1* operon for sequence verification.** L - Quick-Load Purple 2-log DNA ladder. Expected band size: 9066bp. L - Purple 2-log DNA ladder, GC1 and CG2 – PCR with 10ul NEB GC enhancer, HF- PCR without 10 μl GC enhancer. DNA: ATCC 53582. GC enhancer was used to achieve amplification despite presence of GC-rich regions in the target sequence.

**A**

**B**

**Supplementary Figure S2. Percent identity matrices of c-di-GMP phosphodiesterase (PdeA), diguanylate cyclases (Dgc) and β-glucosidase (BglxA).** Amino acid sequences were aligned and percent identity calculated using MUSCLE60, and all gaps were removed from analysis.


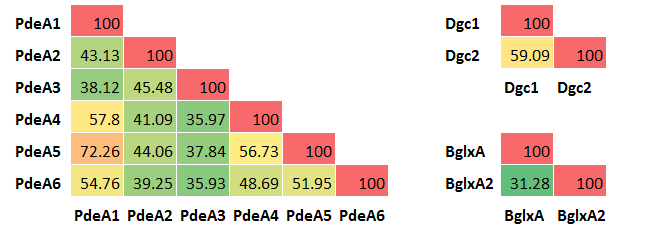


**Supplementary Figure S3. Percent identity matrices of 16s rRNA sequences of ATCC 53582, *G. hansenii* LMG 1527, *G. hansenii* ATCC 23769 and *G. hansenii* RG3.** These strains form a closely related group, with over 99% 16s rRNA sequence identity. Nucleotide sequences were aligned and percent identity calculated using MUSCLE60, and all gaps were removed from analysis.


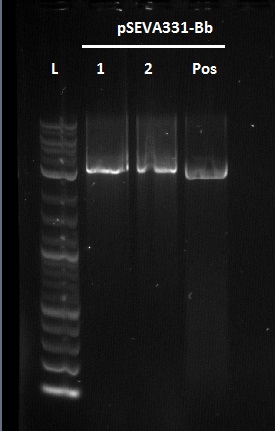


**Supplementary Figure S4. Verification of transformation of *G. hansenii* ATCC53582 with pSEVA331Bb.** To test for the reproducibility of transformation, electrocompetent *G. hansenii* ATCC 53582 cells were prepared and transformed according to the protocol described in *Materials and methods* by a second experimenter. Transformed colonies were inoculated into HS medium, plasmid DNA re-isolated, linearized with NcoI and imaged to confirm transformation and propagation of pSEVA331Bb in ATCC 53582. L – NEB Quick-Load Purple 2-log DNA ladder, 1 and 2 – biological replicates of pSEVA331Bb prepared from *G. hansenii* ATCC 53582, Pos – pSEVA331Bb prepared from *E. coli* Turbo. Note that the genome sequence of *G. hansenii* ATCC 53582 indicates that it harbours no native plasmids around 3kbp in size.

# **Supplementary Tables**

**Supplementary Table S1. Details of plasmids tested for replication in *G. hansenii* ATCC 53582.** Of the 9 tested plasmids, pSEVA331, pSEVA351, pBAV1K and pBla-Vhb-122 were capable of replication in ATCC 53582. However, note that failure to see replication with other plasmids may be attributed to low transformation efficiencies of ATCC 53582.


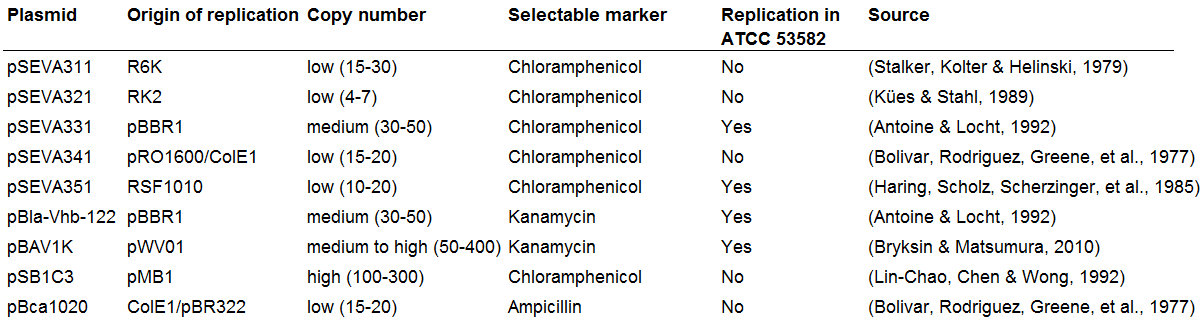


**Supplementary Table S2. Primers used for verification of transformants via colony PCR.**


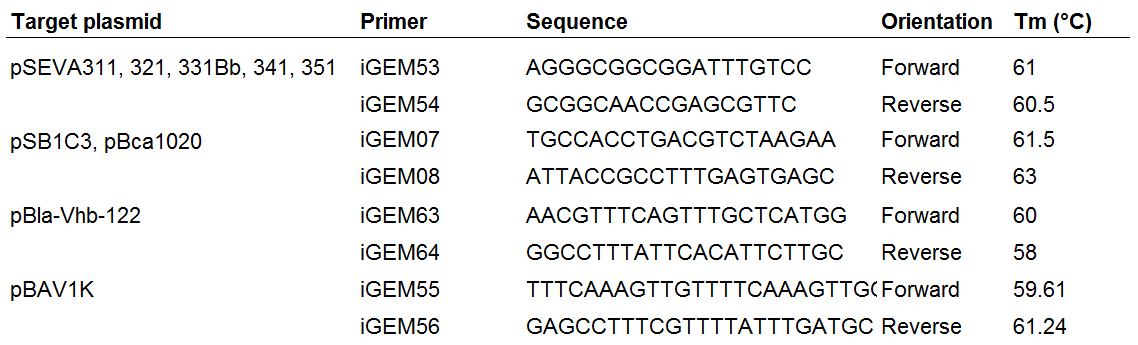


**Supplementary Table S3. Thermocycling programs for colony and long-range PCR.**


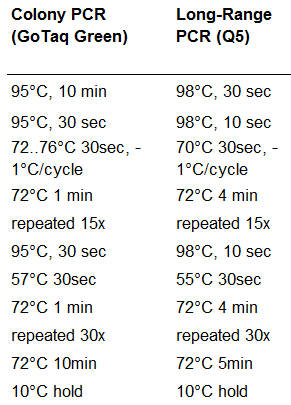


# **Supplementary Methods**

## **Preparation of electrocompetent *G. hansenii* ATCC 53582 cells**

- Materials
  - HS+cellulase media
  - Ice bucket and ice
  - Temperature controlled centrifuge
  - 1mM HEPES (ph7.0) 80 mL
  - 15% glycerol
  - 50 mL tubes
  - P1000 pipette and tips
  - Stripettes and an automatic pipette for larger volumes
  - Shaker at 30 °C, 230 rpm
- Methods

1. Inoculate 5 mL of HS+0.2% (v/v) cellulase medium in 50 mL tubes with *Gluconacetobacter*.
2. Incubate at 30°C, 230 rpm shaking, 45° angle overnight or until cloudy.
3. Next day, pour 10 mL of HS+cellulase medium into each of 8 of 50 mL tubes.
4. To each tube, add seed culture to OD_600_=0.04 and incubate with shaking at 230rpm, 30°C, 45° angle overnight until OD_600_=0.4-0.7. It is important to keep cells in this range, as both too high and too low concentrations result in low transformation efficiencies.
5. Before continuing, set up the necessary materials:
   1. Pre-cool centrifuge to 4°C
   2. Prepare ice bucket
   3. Chill 1mM HEPES buffer and 15% glycerol buffer on ice
   4. Label glycerol stock tubes
6. Once the cultures reach desired OD_600_, take them out of incubation and put them on ice for 10 minutes (the tube should feel cool).
7. From here on, keep cells at 4°C.
8. After cooling, spin the tubes in a refrigerated (4°C) centrifuge for 12min at 3200 g.
9. Pour off supernatant carefully, taking care not to pour off the pellet. *G. hansenii* ATCC 53582 does not pellet as easily as *E. coli*, most likely due to the buffering effects of cellulose. If the pellet is not attached to the wall after centrifugation, smear the pellet onto the wall of the tube and centrifuge again using longer centrifugation times.
10. Re-suspend bacteria in 10 mL HEPES: re-suspend first using 1 mL HEPES and a P1000 pipette, then add 9 mL of HEPES using a stripette; it is much easier to re-suspend the pellet fully using a P1000.
11. At this point can pool 2 samples into one tube to reduce handling time.
12. Centrifuge again for 14 minutes at 3200g or higher and 4 °C temp.
13. Pour off supernatant, re-suspend pellet in 10 mL ice-cold HEPES on ice as before.
14. Centrifuge for 14 minutes at 4100 rpm, 4 °C.
15. Pour off supernatant and re-suspend pellet in 1 mL ice cold 15% glycerol solution. Pool all samples and add glycerol to a total of 6 mL.
16. Pipette 100 μL aliquots into tubes. Store samples on ice for immediate use or freeze aliquots in-80°C. The efficiency of electrocompetent cells may reduce after each freezing, so immediate use may result in highest efficiencies.

## **Transformation of *G. hansenii* ATCC 53582**

- Materials
  - Plasmid DNA
  - Electrocompetent *G. hansenii* cells (100 µL aliquots)
  - Ice bucket and ice
  - 1 mm path electrocuvettes
  - 1.5 mL microcentrifuge tubes or PCR tubes
  - An electroporator set at 3kV and 5.9ms
  - HS+cellulase medium
  - HS agar plates with appropriate antibiotic
  - One aliquot of electrocompetent cells for negative and one for positive control
- Methods

1. Set up the electroporator with settings at **3kV, 5-8ms** – lower voltages seem to reduce transformation efficiencies.
2. Prepare 15 mL culture tubes (Corning) containing 800 µL HS+cellulase media.
3. Prepare ice bucket, place plasmid DNA and electrocuvettes on ice and thaw electrocompetent cells on ice. NB! Make sure DNA is desalinated and pure before use – ionic solutions can cause arcing and impurities can reduce efficiencies.
4. Add 2 µL (20-200ng/μL) of plasmid DNA to 100 µL of concentrated cells in a cold microcentrifuge or PCR tube and mix well by pipetting. Don’t add plasmid DNA to one aliquot of electrocompetent cells for negative control.
5. Transfer the cell/DNA mixture to a cold electroporation cuvette. Dry any water condensate outside of the cuvette, place the cuvette into the electroporator, and apply the pulse.
6. Transfer the pulsed cells into 800 µL of HS+cellulase medium in a culture tube.
7. Incubate the culture tubes with shaking (230 rpm) at 30 °C for approximately **16 hours –** shorter incubation times seem to reduce numbers of transformants.
8. Spin the culture at max available RPM for 10 minutes, resuspend in 200 μL HS and plate on an HS-agar plate with an appropriate antibiotic concentration.
   1. For plasmids pSEVA331Bb and pSEVA351 the appropriate chloramphenicol concentration is 17-34μg/ml and for pBla-VHb-122 and pBAV1k, the kanamycin concentration is 50-100μg/ml. When other backbones are used, antibiotic concentrations may need to be re-evaluated to be suitable for the plasmid.
9. Grow plates at 30°C inverted, colonies should appear in 24-72 hours. With low antibiotic concentrations, false positive colonies without plasmid may be present, therefore it is always necessary to verify the presence of plasmid on the DNA level, using PCR or test digests.
